# Incidence of post-cataract endophthalmitis at Aravind Eye Hospital

**DOI:** 10.4103/0301-4738.71704

**Published:** 2010

**Authors:** Rohit C Khanna, Chandrasekhar Garudadri

**Affiliations:** International Centre for Eye Health, London School of Hygiene and Tropical Medicine, London, U.K; 1 L V Prasad Eye Institute, Hyderabad, India,

Dear Editor,

The recommendations for the practice patterns of cataract surgery in the recently published article by Ravindran *et al*.,[1] appear to offer huge benefits for cost control in cataract surgery. A similar report from the same group in the past was published and debated.[2,3] Going by the Hippocratic oath of “above all do no harm”, if the study methodology and reporting are flawed, these recommendations could potentially result in an increase in the incidence of the most dreaded complication of cataract surgery. Before we consider extrapolating these recommendations, a critical review of the study methodology and the results is mandatory.

The authors claim an endophthalmitis rate of 0.09% in a series of 42,426 patients. It looks like they have included as endophthalmitis only those patients who had an intervention in their retina service. While the protocols for operating room procedures and the experience of the surgeons are reported in detail, equal rigor for postoperative evaluation is absent. In the absence of an accepted definition of endophthalmitis and information about how many of the full time surgeons (FTS) or surgeons in training (SIT) actually evaluated these patients in the postoperative period, along with possible audit or quality control of these evaluations, it is difficult to rely on the reported endophthalmitis rate.

The authors claim that endophthalmitis is more common in manual small incision cataract surgery (MSICS); one could argue from the data that endophthalmitis is more common in the charity patients. Prevalence of endophthalmitis is 3 / 12022 (0.02) in private and 35 / 30404 (0.12) in charity (*P*= 0.0039). The huge loss to follow-up in the charity patients could have led to under-reporting of endophthalmitis. Nearly, one out of every six (16%) of the charity patients are lost to follow-up.

The authors make a presumption that cases that develop complications would come back to their hospital, however, this presumption is not tenable, for two possible reasons. Firstly 75% of the charity patients are outstation patients. The cost of surgery for the outstation patient even in charity (transportation, cost of intraocular lens, cost of attendant travel etc.) is significant. The average poor, old, dependent, rural Indian patient is more likely to resign from seeking further care and attribute the non-recovery of vision to his *“Karma”*. Secondly, on a purely scientific basis one would need to consider the worst case scenario which will increase the endophthalmitis rate to 13.3%.

Lack of rigor in data collection and analysis is also reflected in the fact that the study period includes patients operated from January 2007 to August 2008, with minimum three months follow-up, the last follow-up should then have been on 30 November, 2008. However, the paper was ready before that and was submitted for publication on 28 November, 2008.

Going by their own data that Nocardia is the predominant cause of endophthalmitis, and the source for this organism being soil, the protocol of allowing patients with street clothes etc. might have contributed to the contamination by Nocardia through street clothes and feet of patients and staff.

In summary, we recommend that changing the practice patterns for cataract surgery based on this data, is not appropriate.
